# Establishment and Verification of the Cutting Grinding Force Model for the Disc Wheel Based on Piezoelectric Sensors

**DOI:** 10.3390/s19030725

**Published:** 2019-02-11

**Authors:** Jing Ni, Kai Feng, M.S.H. Al-Furjan, Xiaojiao Xu, Jing Xu

**Affiliations:** School of Mechanical Engineering, Hangzhou Dianzi University, Hangzhou 310005, China; nj2000@hdu.edu.cn (J.N.); fengkai319@foxmail.com (K.F.); xuxiaojiao66@foxmail.com (X.X.); hduxujing@163.com (J.X.)

**Keywords:** Grinding cutting force model, Disc Wheel, Effective grits’ number, Piezoelectric sensors

## Abstract

In this paper, a new model of cutting grinding force for disc wheels is presented. Initially, it was proposed that the grinding cutting force was formed by the grinding force and cutting force in combination. Considering the single-grit morphology, the single-grit average grinding depth, the effective number of grits, and the contact arc length between the grit and the workpiece comprehensively, the grinding force model and the cutting force model were established, respectively. Then, a universal grinding cutting force model was optimized by introducing the effective grit coefficient model, dependent on the probability statistical method and the grit height coefficient model with Rayleigh’s distribution theory. Finally, according to the different proportions of the grinding force and cutting force, the grinding cutting force model, with multi-particles, was established. Simulation and experimental results based on piezoelectric sensors showed that the proposed model could predict the intermittent grinding cutting force well. Moreover, the inclusion of the grit height coefficient and the effective grits number coefficient improved the modeling accuracy. The error between the simulation and experimental findings in grinding cutting force was reduced to 7.8% in comparison with the traditional model. In addition, the grinding cutting force can be divided into three segments; increasing, steadiness, and decreasing, respectively found through modeling.

## 1. Introduction

The grinding wheel machine is widely used in machinery, metallurgy, mining, construction, automobile manufacturing, and other industries. It is possible to cut-off non-metallic materials, metallic materials, and even parts of non-ferrous metals in high rotation speed, continuously, through this process [1]. It is known that grinding cutting is a multi-point cutting process that affects the dimensional accuracy and disc lifetime. Because of the diverse morphology of the disc wheel, the irregular distribution of grits, and the uncertainty of the number of effective grains, it is difficult for the traditional identification methods to calculate the grinding cutting force precisely, smoothly, and efficiently [2,3,4]. Therefore, the grinding cutting load characteristics of the grinding wheel disc in the grinding process and the calculation model of the grinding load of the grinding wheel disc with multiple grinding particles have important theoretical research value and engineering benefits for improving the machining accuracy, production efficiency, and cost involved.

At present, three types of grinding cutting force models have been put forward. The first is a physical grinding model and a numerical simulation model of single-grit. For instance, Duan et al. [5] assumed the shape of the abrasive grain to be a cone and carried out the three-dimensional grinding simulation of the single-grit, which better solved the element distortion in the grinding simulation with the finite element method. Lang et al. [6] assumed the shape of the single-grit to be a conical shape and also assumed that the protrusion height obeyed the Rayleigh distribution, thus they derived the formula of the thickness of the undeformed abrasive chip. Su et al. [7,8] obtained the stress variation of the grits and the workpiece material by simulating a single abrasive cutting process and analyzed the wear mechanism of the grits and the grinding deformation mechanism of the workpiece material. Yan et al. [9] simulated the single-grit grinding process with the finite element method (FEM)-software AdvantEdge, established a mechanical model and numerical simulation model of a single-grit grinding under different process parameters, and verified the proposed model with scratch tests under different loads.

The second kind of grinding cutting force model is a statistical analysis of multi-particle effective grinding force. For instance, Engin et al. [10] used multiple regression analyses for circular sawing (CS) and abrasive water jet cutting (AWJC) and created a predictive chart of specific energy for the shore hardness and wear resistance. Ersoy et al. [11] successfully used statistical multivariate linear regression analysis techniques to evaluate the combined factors of the wear performance on grinding. Karakurt et al. [12] established the calculation formula of the grinding force by quoting the multi-factor statistical method and considering the grinding speed, grinding depth, and other related factors. Turchetta [13] performed a linear regression analysis on the experimental data of grinding. The results show that the radial cutting force increases with an increase in feed speed and the tangential cutting force increases with an increase in the equivalent cutting thickness. Zhang et al. [14] assumed the grits to be of a normal distribution and calculated the number of grains in the surface of the disc wheel, establishing a theoretical calculation model of the grinding force. Based on the theoretical model of the grinding force on the single-grit surface of a diamond grinding wheel, Agarwal and Rao [15,16] and Hecker et al. [17] considered the law of the protrusion height on the grit grain to be in accordance with the Rayleigh distribution and established a mathematical model for predicting the grinding force of multiple grit grains. In order to determine the actual number of grits per unit area and the number of grits per unit area engaged in grinding, Hou and Komanduri [18] proposed probabilistic methods to analyze the grinding process.

The third kind of grinding cutting force model is a simulation of multi-particle grinding forces under a complex array. Su et al. [19] proposed that all grits with the same diameter were distributed randomly and established the grinding force calculation model of a two-dimensional disc wheel. Hegeman [20] optimized the grinding process, simplified the grits into elliptic grits, and established a three-dimensional grinding wheel model. Then, simulations of the grinding force for two different materials (cemented carbide and manganese zinc ferrite) were carried out to verify the effectiveness of the cutting model. Warnecke and Barth [21] unified the grinding wheel and the workpiece into a finite element model for simulation, studied the influence of the grinding wheel vibration on material removal, and established the grinding force load calculation model. Su et al. [22] used a regular hexahedron as the basic form of grits and randomly distributed the grits on the grinding wheel to establish a grinding simulation model.

However, in the current literature, the morphology of single-grit has not been accurately considered in the establishment of the grit grinding force model. On the other hand, the number of effective grains and the unequal height arrangement of the grits on the disc wheel were also not well thought out when establishing the multi-particle grinding force model. Thus, further study is still needed to determine the multi-particle grinding cutting force model precisely. In this paper, the morphology of the grinding grit is simplified to rigid hexahedrons and a new model of multi-particle grinding cutting force is presented by considering the single-grit morphology, cutting depth, contact arc length, and number of effective grits. The simulation and experimental results with piezoelectric sensors showed that the obtained model is simple, effective, and versatile.

## 2. Mechanistic Model of the Cutting Grinding Force

### 2.1. Analysis of the Grinding Cutting Force

**Figure 1:**
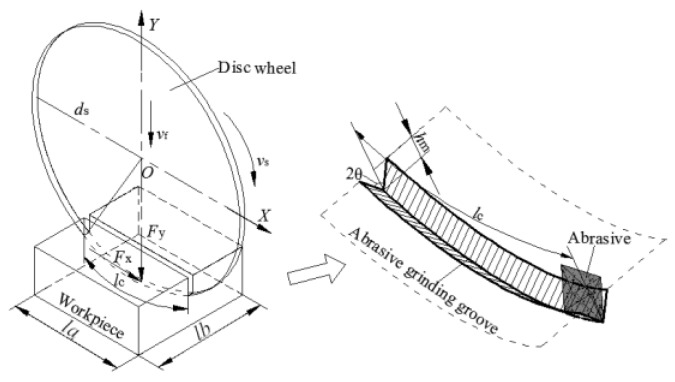
The grinding principle of the disc wheel.

The grinding cutting force is a physical phenomenon caused by the contact of a grinding edge and a workpiece. Figure 1 shows the grinding cutting principle of a disc wheel, where *d_s_* is the disc diameter, *v_s_* is the grinding cutting speed, *v_f_* is the feed speed, *h_m_* is the average comprehensive grinding depth of single-grit, *l_c_* is the contact arc length, *θ* is the grinding angle, *l_a_* is the workpiece length, and *l_b_* is the workpiece width. As shown in Figure 1, the grinding cutting force of the disc wheel includes *F_X_*, *F_y_*, and *F_z_,* where *F_X_* is the most important component. Thus, this paper mainly focuses on the horizontal grinding cutting force (*F_X_*).

According to hundreds of grinding cutting experiments, the following points have been identified:

(1) The grinding cutting force is formed through the combination of “grinding force” and “cutting force” and the proportions are different.

(2) The grinding force emerged through the simultaneous action of the grits; additionally, every single-grit could be regarded as a rigid polyhedron.

(3) The single-grit average grinding depths were extremely thin and different from each other.

(4) Some grits have involvement in grinding, but just above the grinding surface.

(5) The grinding force is added to the increase in the contact arc length between the grit and the workpiece.

Thus, the grinding cutting process was extremely sophisticated. In addition, the modeling process not only needed to consider the characteristics of the grinding force and cutting force, but also the grinding edge shape, the single-grit average grinding depth, the number of effective grains, and the contact arc length between the grit and the workpiece. Hence, this paper established the grinding force model (*F_X_*_1_) and cutting force model (*F_X_*_2_), respectively, and, according to the different proportions of the grinding force and cutting force, the grinding cutting force model with multi-particles was established.

### 2.2. Computational Model of the Grinding Force

As shown in Figure 1, the essence of the grinding force could be regarded as a common grinding action of many single-grits with micro edges. The grinding force (*F_X_*_1_) could be regarded as the total sum of the single-grit grinding forces (*f_X_*_1_) simultaneously involved in grinding on the surface of the disc wheel. Thus, the *F_X_*_1_ could be expressed as follows:

(1) $$F_{X1}=n_{e}\;.\;f_{X1}$$

where *n_e_* is the number of effective grains in the contact arc zone and *f_X_*_1_ is the single-grit grinding force.

The single-grit grinding *f_x_*_1_ has the following relationship with the grinding area of the bilateral surface (*A_g_*) [23]:

(2) $$f_{X1}=k_{s1}\cdot {\left(A_{g}\right)}^{\gamma }$$

where *k_s_*_1_ is the specific pressure associated with the grinding material, *A_g_* is the grinding area of a bilateral surface of the single-grit, and *γ* is a dimensionless constant.

According to the geometric relation of Figure 1, *A_g_* could be expressed as:

(3) $$A_{g}=\frac{h_{m}\cdot l_{c}}{\cos\theta }$$

where *θ* is the grinding angle, *h_m_* is the average comprehensive grinding depth of the single-grit, and *l_c_* is the contact arc length. 

Thus, after comprehensive consideration of Equations (1)–(3), the grinding force foundation model could finally be expressed as follows:

(4) $$F_{X1}=K_{s1}\;.\;{\left(\frac{h_{m}.l_{c}}{cos\theta }\right)}^{\gamma }\;.n_{e}$$

Therefore, as seen in formula (4), we obtain the exact values of *θ*, *h_m_*, *l_c,_* and *n_e_*. Thus, the *F_X_*_1_ will be calculated.

(1) The Model of the Grinding Angle (*θ*):

The grinding process is a physical phenomenon caused by the contact between the grinding edge and materials. Its essence could be regarded as the common grinding action of a large amount of single-grit with a micro edge. However, due to the diversity of the grit morphology in the surface of the disc wheel, it is difficult to calculate the grinding angle (*θ*) in the modeling of the grinding force characteristics. Therefore, it is necessary to simplify the grit morphology to determine the grinding angle (*θ*).

**Figure 2:**
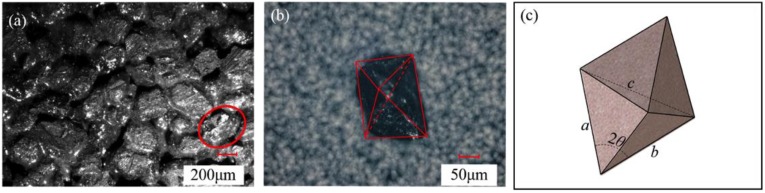
The principle diagram of the simplification of single-grit: (**a**) The surface of the disc wheel; (**b**) Single-grit morphology; (**c**) The single grain model.

Figure 2 represents the grit morphology screened by ultra-deep microscope photography. As in Figure 2a, grits in the disc wheel surfaces are in an irregular arrangement with multiple edges. Fortunately, as shown in Figure 2b, most grits are a polyhedron with a clear edge contour under the observation of single-grit peeled off from the disc wheel, which looks like a hexahedron. Regardless of whether the grinding grains are tetrahedral or hexahedral, the actual sharpening edges are the corners of the grinding grain. Therefore, as in Figure 2c, the grinding grains are simplified into rigid hexahedrons and each length of hexahedron is measured and counted with many grits to determine the grinding angle (*θ*), as shown in Table 1. Lastly, the grinding angle is calculated using the cosine theorem (Equation (5)). The grinding angle (*θ*) was calculated as 36.87.

(5) $$\cos(2\theta )=\frac{a^{2}+b^{2}-c^{2}}{2ab}$$

**Figure 3:**
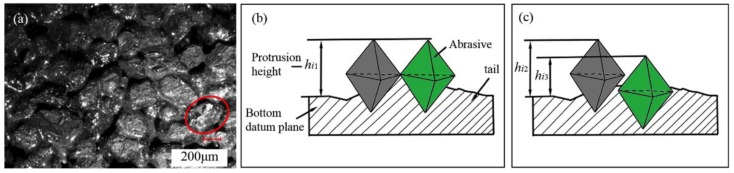
The protrusion height of the grits: (**a**) The surface of the disc wheel; (**b**) An equal height grit [24]; (**c**) An unequal height grit.

(2) The Model of the Comprehensive Grinding Depth of Single-grit (*h_m_*):

Figure 3 shows the protrusion height of the grits, whereas the existing research studies have treated the single-grit average grinding depth as uniform, as shown in Figure 3b. It can be written as follows [24]:

(6) $$h_{m}=\frac{18\cdot \pi d_{s}v_{f}}{5\cdot b_{w}n_{t}v_{s}}$$

where *n_t_* is the total grinding grain number in a grinding surface and *b_w_* is the blade thickness.

**Figure 4:**
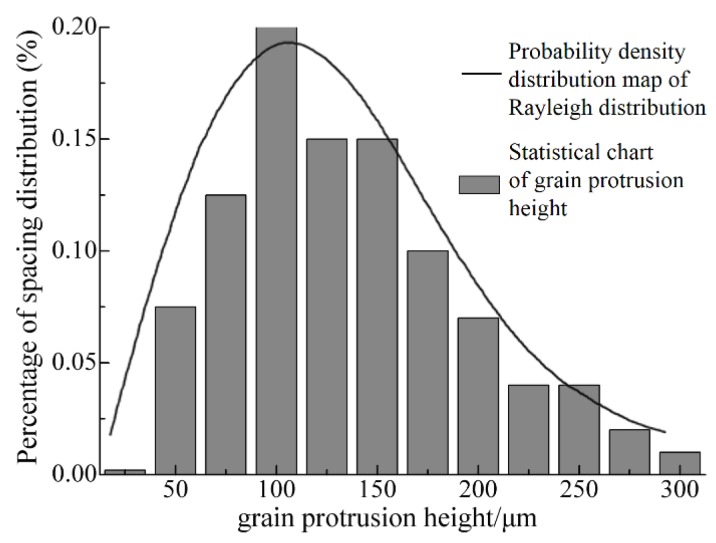
A diagram of the grain protrusion height.

However, a large number of observations indicated that the grits were of uneven height on the disc surface, as shown in Figure 3c. Therefore, the above calculation has an error and it is necessary to optimize the above model. As shown in Figure 4, we found that the statistical chart of the grit protrusion height, counted with hundreds of measurements, is very similar to the probability density distribution map of Rayleigh’s distribution with the observation and sampling by a microscope with depth. Therefore, the Rayleigh distribution could be used to explain the protrusion height of single-grit. Its function can be described as follows:

(7) $$f(h)=\{\begin{array}{cc} \frac{h}{\sigma^{2}}\cdot e^{-\frac{h^{2}}{2\sigma^{2}}} & h\geq 0\\ 0 & h<0 \end{array}$$

where *h* is the grit protrusion height and *σ* is the probability density function parameter (*σ* = 100 μm here), as seen in Figure 4.

The grit height coefficient is introduced by the Rayleigh distribution to optimize the grinding depth (*h_m_*). As shown in Figure 4, the grain protrusion height ranges from 50 μm to 300 μm. Using the Newton-Leibniz formula and the probability density calculation, the grit height coefficient (*ε*) can be expressed as:

(8) $$\varepsilon =F(h)=\int {}_{50}^{300}f(h)dh$$

where *ε* is the grit height coefficient.

From Equations (6) and (8), the single-grit average grinding depth (*h_m_*) can be written as follows:

(9) $$h_{m}=\varepsilon \cdot \frac{18\cdot \pi \cdot d_{s}v_{f}}{5\cdot b_{w}n_{t}v_{s}}$$

**Figure 5:**
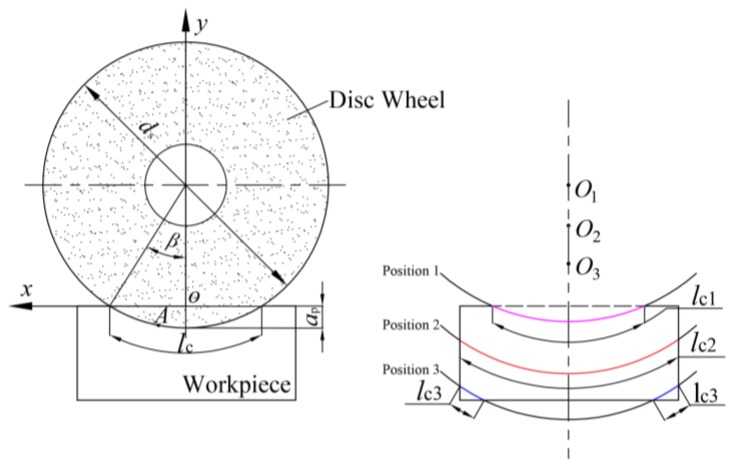
The motion track and contact arc length of the grits in grinding.

(3) The Model of the Contact Arc Length (*l_c_*):

Figure 5 shows the grit trajectory and contact arc length, where *β* is the workpiece contact angle, *a_p_* is the linear displacement related to the workpiece, and when the blade turns angle *β*, *l_c_*_1_ is the contact arc length in the initial phase with the center disc wheel at position O_1_, *l_c_*_2_ is the contact arc length in the intermediate stages with the center disc wheel at position O_2_, and *l_c_*_3_ is the contact arc length in the final stages with the center disc wheel at position O_3_. It is assumed that grit A on the disc surface is in the beginning of the workpiece, connected with the blade. That blade is performing a feed movement in the vertical direction. In the coordinate plane XOY, the trajectory equation of grit A can be written as follows:

(10) $$\{\begin{array}{c} x=\frac{d_{s}}{2}\cdot \sin\beta \\ y=\frac{d_{s}}{2}\cdot \left(1-\cos\beta \right)-a_{p} \end{array}$$

Here, *a_p_* can be expressed as $a_{p}=\frac{v_{f}\cdot d_{s}}{4\pi \cdot v_{s}}\beta$, and the *a_p_* value can then be substituted into Equation (10) and derivated:

(11) $$\{\begin{array}{c} dx=\frac{d_{s}}{2}\cdot \cos\beta d\beta \\ dy=\left(\frac{d_{s}}{2}\cdot \sin\beta -\frac{v_{f}\cdot d_{s}}{120\cdot v_{s}}\right)d\beta \end{array}$$

The differential value of the contact arc length (*l_c_*) can be calculated by Equation (11):

(12) $$dl_{c}=2\sqrt{\frac{d_{s}{}^{2}}{4}+{\left(\frac{v_{f}\times d_{s}}{120\times v_{s}}\right)}^{2}}d\beta$$

By integrating Equation (12), the contact arc length can be written as follows:

(13) $$l_{c}=4\sqrt{\left[\frac{d_{s}{}^{2}}{4}+{\left(\frac{v_{f}\cdot d_{s}}{120\cdot v_{s}}\right)}^{2}\right]\left(\frac{a_{p}}{d_{s}}\right)}$$

Without considering the influence of the feed movement (*v_f_* = 0), the contact arc between the disc wheel and the workpiece can be expressed as:

(14) $$l_{c}=\{\begin{array}{cc} 2\cdot \sqrt{d_{s}\cdot a_{p}} & 0\leq a_{p}\leq p_{s}\\ d_{s}\cdot \arcsin\frac{l_{b}}{d_{s}} & p_{s}\leq a_{p}\leq b\\ d_{s}\cdot \arcsin\frac{l_{b}}{d_{s}}-2\cdot \sqrt{d_{s}\cdot a_{p}} & a\leq a_{p}\leq a+p_{s} \end{array}$$

where *p_s_* is the depth when the disc wheel contacts the workpiece side immediately and can be expressed as:

(15) $$p_{s}=\frac{d_{s}-\sqrt{d_{s}{}^{2}-l_{b}{}^{2}}}{2}$$

Equation (15) is the contact arc length between the disc and the workpiece in the intermediate stages of the inception phase and the final stages.

**Figure 6:**
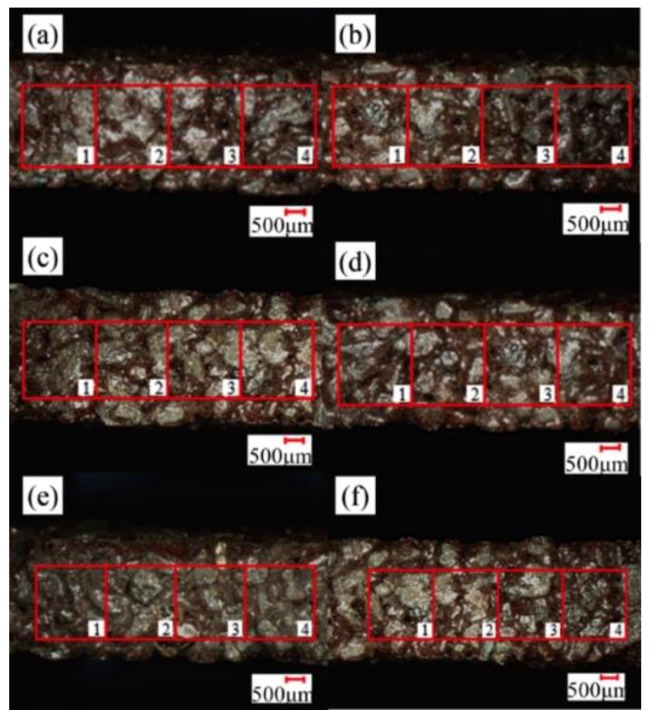
A microscopic amplification diagram of the grinding wheel. (**a**) Area a; (**b**) Area b; (**c**) Area c; (**d**) Area d; (**e**) Area e; (**f**) Area f.

(4) The Model of the Number of Effective Grains:

(a) Computing method of the grit density:

A probability statistical method (sampling distribution) is used to calculate the grit density. Firstly, the disc surface is divided into several square areas (*s*_1_) equally and as a whole (*N*). Secondly, several random regions (*k*), (*X*_1_, *X*_2_, …, *X_k_*), have been extracted, leaving a random sample with a capacity of *K* from the total *N*. Figure 6 shows the six different areas enlarged in the same new blade, numbered 1, 2, 3, 4, 5, and 6. Four regions in each picture (*s*_1_ = 4 mm^2^, *k* = 24) are selected. Thus, the grit density (*ρ_s_*) could be expressed as:

(16) $$\rho_{s}=\frac{\sum_{i=1}^{k}X_{k}}{k\cdot s_{1}}$$

The grit density, counted by a stereomicroscope, was added up in Table 2. The quantity of the grit in every area varies from 3 to 6 and the grit density remains from 1 to 1.31. Thus, according to the calculation of Equation (16), the grit density (*ρ_s_*) is 1.16 mm^−2^.

(b) Computing method of the number of effective grains:

Firstly, the total number of grits (*n_t_*) in effective contact arc areas could be expressed as:

(17) $$n_{t}=l_{c}\times b_{w}\times \rho_{s}$$

**Figure 7:**
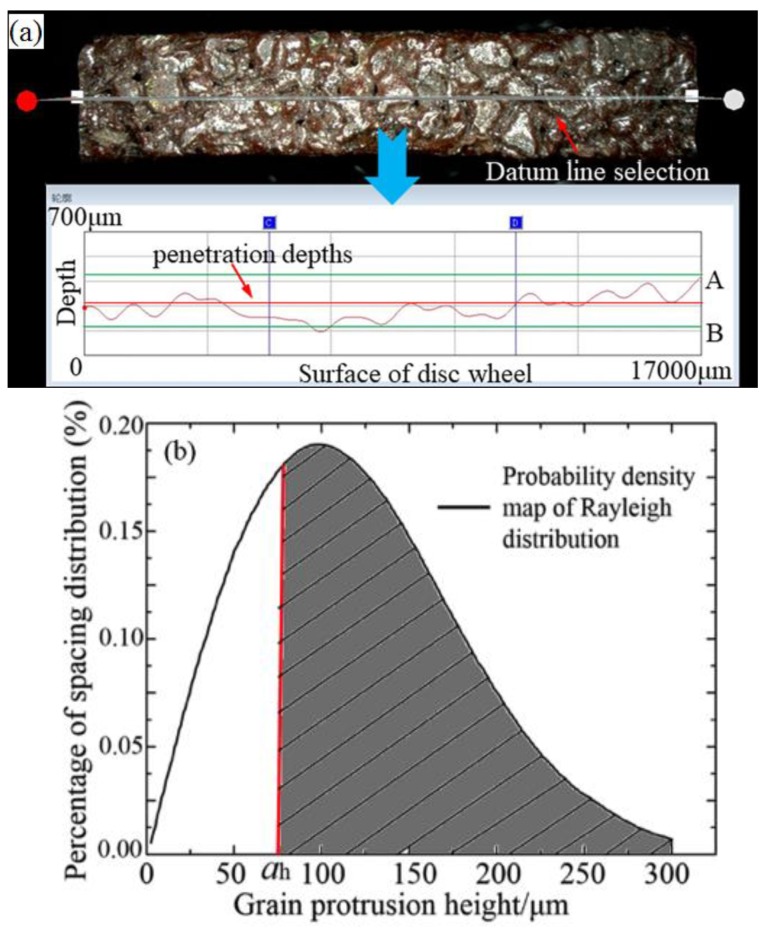
The bulge height distribution diagram of the surface grit particle in the grinding wheel: (**a**) The bulge height distribution diagram; (**b**) The position of the penetration depths.

Secondly, the grits in the disc surface are uneven and some grits’ grinding edges were on the disc surfaces while others were embedded at a certain depth, as shown in Figure 7a. Therefore, the number of grits presented in grinding was less than that on the disc wheel surface. Additionally, it is necessary to count the actual grits involved in the grinding.

**Figure 8:**
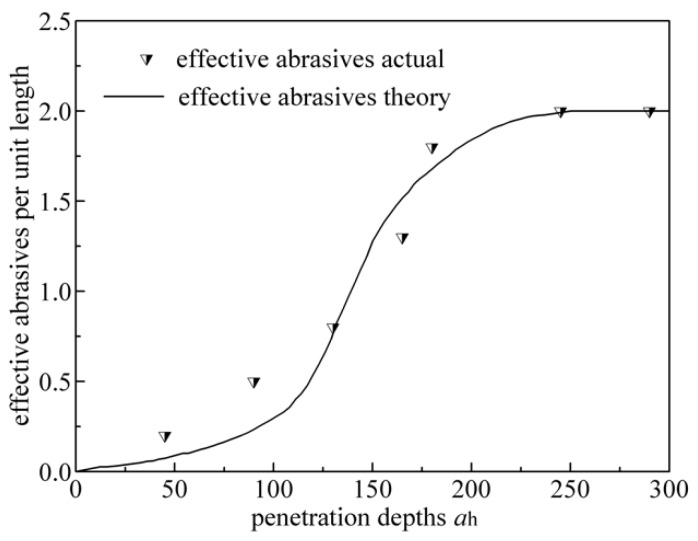
The relationship between the penetration depths and the effective grits per unit length [25].

Using the analysis methods of Zhang [25], the grits on the work surface of the disc wheel are uneven. If we determined a certain penetration depth along the disc wheel radius (*a_h_*), it could be regarded as being involved in grinding. Figure 8 shows the relationship between the penetration depths and the effective grits per unit length.

As seen from Figure 8, the greater the penetration depth, the greater the number of effective grits, however, when the penetration depth increases to a certain extent, the number of grinding edges will not increase any more. Based on analysis and deduction in [25], the penetration depths can be expressed as:

(18) $$a_{h}={\left[\frac{2}{C_{1}K_{s}}\right]}^{\frac{1}{p+1}}{\left[\frac{v_{f}}{v_{s}}\right]}^{\frac{1}{p+1}}{\left[\frac{a_{p}}{d_{eq}}\right]}^{\frac{1}{2(p+1)}}$$

where *a_h_* is the penetration depth, *d_eq_* is the equivalent diameter of the disc wheel, *C*_1_ is the coefficient related to the density of the grinding edge, *K_s_* is the coefficient related to the shape of the grinding edge, and *p* is the index (*p* = 2).

Figure 8 represents the probability of the density distribution map of the Rayleigh distribution and shows that a small part of the grits is not involved in grinding, with the calculation of Equation (18). Therefore, the effective number of grains can be optimized by introducing an effective grit coefficient (*η*). By combining the computing method of the penetration depths, the effective grit coefficient (*η*) can be expressed as:

(19) $$\eta =\int_{a_{h}}^{300}f(h)dh$$

Finally, by considering Equations (17) and (19) comprehensively, the effective number of grains (*n_e_*) can be expressed as:

(20) $$n_{e}=\eta \cdot l_{c}\cdot b_{w}\cdot \rho_{s}$$

(5) A computational model of the multi-particle grinding force:

This yields the equations cited elsewhere (1), (9), (14), and (20). The computational model of the multi-particle grinding force can be expressed as:

(21) $$F_{X1}=\{\begin{array}{cc} k_{s1}\cdot {\left(\frac{\varepsilon d_{s}v_{f}l_{c1}}{b_{w}n_{t}v_{s}}\right)}^{\gamma }\cdot \eta \cdot n_{t} & 0\leq a_{p}\leq p_{s}\\ k_{s1}\cdot {\left(\frac{\varepsilon d_{s}v_{f}l_{c2}}{b_{w}n_{t}v_{s}}\right)}^{\gamma }\cdot \eta \cdot n_{t} & p_{s}\leq a_{p}\leq a\\ k_{s1}\cdot {\left(\frac{\varepsilon d_{s}v_{f}l_{c3}}{b_{w}n_{t}v_{s}}\right)}^{\gamma }\cdot \eta \cdot n_{t} & a\leq a_{p}\leq a+p_{s} \end{array}$$

The Equation (21) is the computational model of the multi-particle grinding force, including the grit height coefficient (*ε*) and the effective grit grain coefficient (*η*).

### 2.3. Computational Model of the Cutting Force

As is known from the traditional theory of cutting force [1], the cutting force in the direction of the disc wheel is related to the cross-sectional area of the chip before plastic deformation and the specific pressure of the workpiece. The model of the cutting force (*F_X_*_2_) could be expressed as follows:

(22) $$F_{X2}=k_{S2}\cdot A^{\beta 1}$$

where *k_s_*_2_ is the specific pressure associated with the grinding material, *A* is the grinding area of the bilateral surface, *β1* is a dimensionless constant, and *A* is related to the adjacent grit spacing (*L*) and the blade thickness (*b_w_*).

(23) $$A=b_{w}\times v_{f}\times \frac{2L}{v_{c}}$$

### 2.4. Computational Model of the Cutoff Grinding Force

Summarizing the above modeling analysis of the grinding force and cutting force, the mathematical model of the wear cutting load characteristic is as follows:

(24) $$F_{X}=\alpha F_{X1}+\left(1-\alpha \right)F_{X2}$$

where *α* is the ratio coefficient of the grinding and cutting characteristics determined by the experimental data.

Based on hundreds of actual grinding cutting test conditions, *α* is a parameter related to the total number of grits (*n_t_*) and the number of effective grains (*n_e_*) on the surface of the disc wheel. Thus, according to the numerical simulation fitting, *α* could be expressed as:

(25) $$\alpha =\exp\left[-\frac{n_{g}}{n_{t}}\right]$$

## 3. Experiment

**Figure 9:**
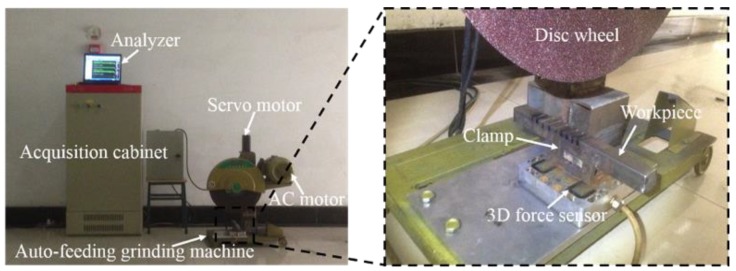
The experimental set-up used for the grinding tests.

Figure 9 shows the auto-feeding grinding machine that was used in the experiment. The necessities of grits include brown alumina (GZ), 24 mesh granularity, a resin binder, a hardness rating (Y), and a straight wheel shape. In our research, the most common material, carbon steel (Type Q235), was used as a workpiece. In order to measure the grinding force, a three-dimensional force piezoelectric sensor (ME K3D120), mounted on the table of the disc wheel with a precision of 0.1% and a measurement range of ±1 kN, was utilized. A clamp to hold the workpiece was bolted onto the dynamometer. The grinding cutting force signal from the sensor was collected by the acquisition instrument (INV3018CT) and analyzed by the software (CIONV DASP V10). Additionally, the sampling frequency was 1 kHz. Further details of the experimental parameters are shown in Table 3. Based on the linear regression analysis and simulation results showed in the Equations (1), (19), and (20), *k_s_* = 0.8, *k_s_*_1_ = 0.92, *k_s_*_2_ = 1.47, and *x* = 0.3365.

## 4. Results and Discussion

**Figure 10:**
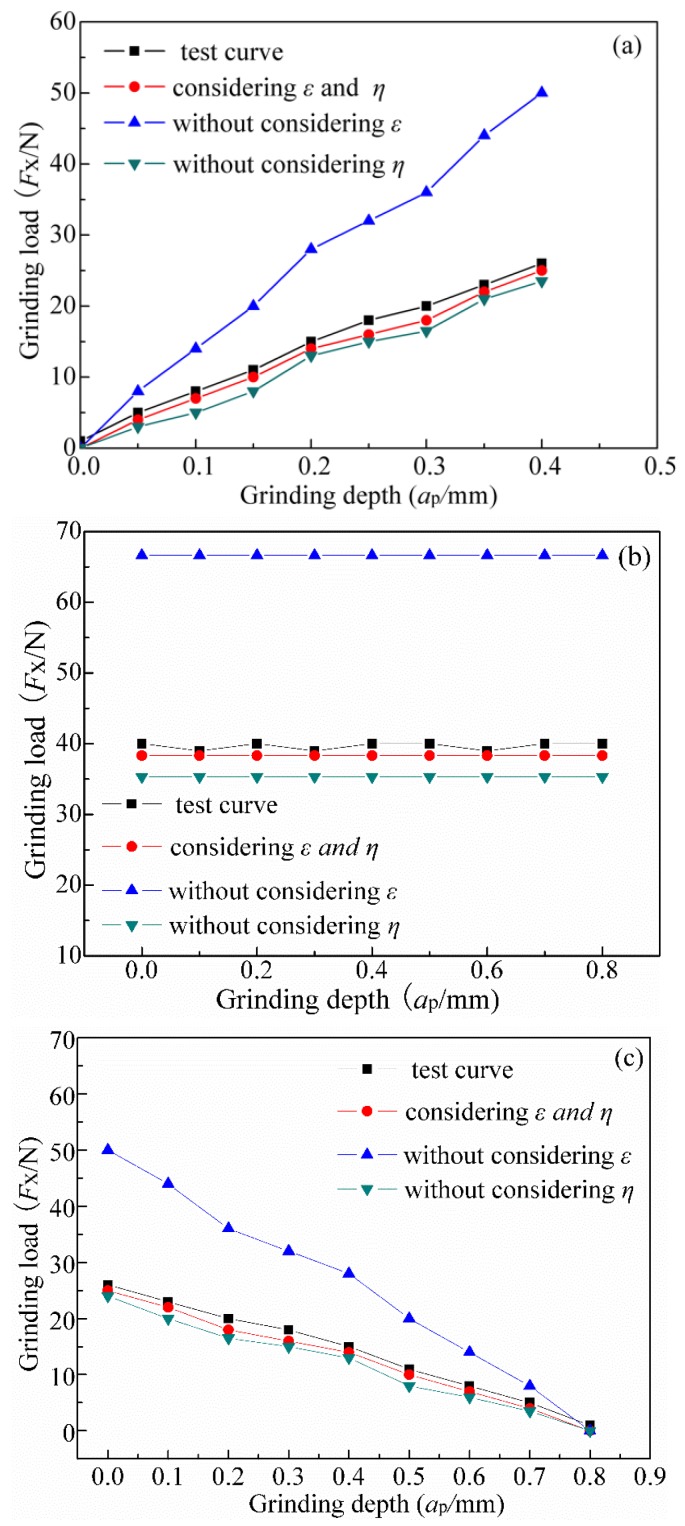
The simulation and experimental horizontal grinding cutting force (*F*_x_): (**a**) Initial stage; (**b**) Intermediary stage; (**c**) Final stage. *ε =* the grit height coefficient and *η* = the effective grit coefficient.

Figure 10 shows the experimental and simulation results (*v_f_* = 0.42 mm/s, *v_s_* = 40 m/s).

(1) As shown in Figure 10a, in the initial phase, the grinding cutting force increased when the number of grits engaged in the grinding process increased. In the intermediate phase, the grinding cutting force remained invariable with an increase in the grinding depth (Figure 10b) because, when the grinding depth increases, the contact arc length (*l_c_*) remains constant, and the effective number of grains remains static, as does the grinding cutting force. Figure 10c shows the final stage, whereas the grinding cutting force decreased with the increase of the grinding depth because, with the grinding depth increasing, the contact arc length (*l_c_*) decreased and the effective number of grains decreased, as well as the grinding force.

(2) On one hand, if we don’t consider the grit height, the coefficient *ε* would improve the model error. This is because the grits in the grinding area are of unequal distribution and the average comprehensive grinding depth of single-grit *h_m_* is not identical. A conclusion that the grain protrusion height had a large influence on establishing the grinding cutting force calculation model could be drawn. The maximum difference of error between the experiment and simulation was 20%, while the average relative error was 13.6%. On the other hand, it could improve the accuracy of the grinding cutting force model by considering the effective grit coefficient (*η*). The maximum difference of error between the experiment and simulation was 15.6% and the average relative error was 13%. The reason for this is that some grains were edged on the disc surface, while others were embedded at a certain depth, which means that the actual number of grinding particles involved in grinding was less than the grits in the disc surface. As shown in Figure 10, the model considering the grit height coefficient (*ε*) and the effective grit coefficient (*η*) had the best agreement with the experimental results. The maximum relative error between the simulation and the experiment was 10% and the average relative error was 7.8%. The main reasons for the error were the following two points: Firstly, due to the non-linear vibration of the machine, the experiment had some deviation and the grinding cutting force became larger. Secondly, the increasing of the chip increased the grinding cutting force during the grinding process. Above all, the conclusion may be drawn that considering the grit height coefficient (*ε*) and the effective grit coefficient (*η*) could improve the calculation accuracy.

## 5. Conclusions

(1) Proposing that the grinding cutting force is formed by a grinding force and a cutting force simplified the grits as rigid polyhedrons, introduced the grit height coefficient, and combined the contact arc length. This paper proposed and optimized the basic model of the grinding cutting force and reduced the simulation error by 10%.

(2) Based on the probability statistical method, which induced the effective grit coefficient and considered the number of effective grains synthetically, this paper established the multi-particles grinding cutting force calculation model and reduced the simulation error by 5.6%.

(3) The simulation and experimental results showed that it could be more precise to establish the multi-particles grinding cutting force calculation model by considering the grit height coefficient and the effective grit coefficient. The simulation and experimental error was not more than 7.8%. Therefore, the model precisely reflected the actual grinding process and provided good theoretical support of technical guidance for the optimization of the process. 

## Figures and Tables

**Table 1:** The size of the grit particles.

No.	a (mm)	b (mm)	c (mm)	Grinding Angle (θ (degree))
1	0.3	0.25	0.25	
2	0.25	0.3	0.35	
3	0.3	0.3	0.27	
4	0.31	0.34	0.35	
5	0.25	0.30	0.28	
6	0.34	0.36	0.37	
Average value	0.29	0.31	0.31	36.87

**Table 2:** The grit number in the disc wheel surface.

Area	Grit Number				Average Number of Grit	Grit Density
	1	2	3	4		
a	6	5	4	6	5.25	1.31
b	4	4	5	5	4.5	1.13
c	3	5	5	5	4.5	1.13
d	6	6	5	4	5.25	1.31
e	3	4	4	6	4.25	1.06
f	5	4	3	4	4	1

**Table 3:** The experimental parameters.

Parameters	Value	Unit
Grinding machine power	3000	W
Rated voltage	220	V
Rated frequency	50	Hz
Blade diameter, *d_s_*	0.4	m
Blade thickness, *b_w_*	0.0032	m
Grinding speed, *v_s_*	40, 45, 50, 55, 60	m/s
Feed speed, *v_f_*	0.42, 0.83, 1.25, 1.67	×10^3^ m/s
Workpiece length, *l_a_*	0.05	m
Workpiece width, *l_b_*	0.05	m
Workpiece height	0.02	m

## Nomenclature

*F_X_*	Cutoff grinding force
*F_X_*_1_	Grinding force
*F_X_*_2_	Cutting force
*f_X_*_1_	Single-grit grinding force
*h_m_*	The average comprehensive grinding depth of single-grit
*l_c_*	The contact arc length
*θ*	Grinding angle
*α*	Ratio coefficient of grinding and cutting
*v_f_*	Feed speed
*v_s_*	Grinding cutting speed
*n_e_*	Effective grain number
*n_t_*	Total grinding grain number
*A_g_*	Bilateral surface
*b_w_*	Blade thickness
*h*	Grit protrusion height
*σ*	Probability density function parameter
*ε*	Grit height coefficient
*a_h_*	Penetration depths
*d_eq_*	Equivalent diameter
*C*_1_	Coefficient related to the density of grinding edge
*K_s_*	Specific pressure associated with grinding material
*k_s_*_2_	Specific pressure associated with cutting material
*β1*	Dimensionless constant
